# Microstructure and Mechanical Properties of Carbides Reinforced Nickel Matrix Alloy Prepared by Selective Laser Melting

**DOI:** 10.3390/ma14174792

**Published:** 2021-08-24

**Authors:** Tian Xia, Rui Wang, Zhongnan Bi, Rui Wang, Peng Zhang, Guangbao Sun, Ji Zhang

**Affiliations:** High-Temperature Materials Department, Central Iron & Steel Research Institute, Beijing 100081, China; xiatian@cisri-gaona.com.cn (T.X.); wangrui@cisri-gaona.com.cn (R.W.); 13617693657@163.com (P.Z.); gbsun611@foxmail.com (G.S.); zhji@ihw.com.cn (J.Z.)

**Keywords:** nickel matrix composite, selective laser melting, nano-carbide particles, microstructure, mechanical properties

## Abstract

Selective laser melting was used to prepare the ceramic particles reinforced nickel alloy owing to its high designability, high working flexibility and high efficiency. In this paper, a carbides particles reinforced Haynes 230 alloy was prepared using SLM technology to further strengthen the alloy. Microstructures of the carbide particles reinforced Haynes 230 alloy were investigated using electron microscopy (SEM), electron probe microanalysis (EPMA) and transmission electron microscopy (TEM). Meanwhile, the tensile tests were carried out to determine the strengths of the composite. The results show that the microstructure of the composite consisted of uniformly distributed M_23_C_6_ and M_6_C type carbides and the strengths of the alloy were higher than the matrix alloy Haynes 230. The increased strengths of the carbide reinforced Haynes 230 alloy (room temperature yield strength 113 MPa increased, ~ 33.2%) can be attributed to the synergy strengthening including refined grain strengthening, Orowan strengthening and dislocation strengthening.

## 1. Introduction 

The Haynes 230 alloy was an advanced solid solution strengthened nickel-based superalloy that contains Cr and W elements as its primary alloying elements [1]. Haynes 230 possesses high-temperature strength, high-oxidation resistant and can be worked in the temperature range between 1198 and 1448 K (925 and 1175 °C) [2]. The weldability and fabricability of Haynes 230 were excellent, and it is being considered for applications including gas turbine hot ending parts, such as heat exchangers, industrial furnace fixtures and muffles. Furthermore, the solid solution strengthened Haynes 230 does not require an age-hardening to precipitate the γ′, which allows Haynes 230 to be applied at a higher temperature, where γ′ strengthened nickel-based superalloys will lose strength due to the solution of γ′ [3]. However, the pure Haynes 230 alloy gradually cannot meet the needs of industrial production due to its limited strengths [4]. Carbide particle reinforced Ni superalloys show great potential for industrial applications by virtue of a high-cost performance ratio, workability and non-polluting properties [5,6,7]. The carbide particle reinforced Ni superalloys are processed by powder metallurgy or casting, which shows the favorable flexibility of the achievable compositions [7,8]. However, the insufficient densification response and inhomogeneous microstructures are most likely to appear in these traditionally manufactured materials [9].

Additive manufacturing (AM) was an advanced technology that was a breakthrough in removal manufacturing by the concept of fabricating the parts directly from powder or wire materials [10,11,12,13,14]. The AM process can produce dense metal parts directly from feedstocks in a layer-by-layer manner with the designed computer-aided design data, which gives AM a fancy ability to fabricate parts without post mechanical processing [15]. Among the several AM technologies, selective laser melting has drawn much attention due to its ability to produce high precision metal parts [16,17,18]. Furthermore, it is capable of achieving the production of parts with complex shapes [19,20]. Hence, SLM was very suitable to prepare high-performance complex superalloy parts. However, the wettability and the bonding of the ceramic particles and the Ni matrix might cause some defects and even premature failure during the mechanical loading [5,8,21,22]. Researchers are still required to clarify the microstructure evolution and mechanical response between Haynes 230 and the in situ carbides reinforced Haynes 230 alloy (CRHA).

In this work, Haynes 230 and the CRHA were successfully processed using SLM. The microstructure difference between Haynes 230 and the CRHA was investigated. The mechanical properties of Haynes 230 and the CRHA were tested and the underlying strengthening mechanisms of the CRHA were analyzed.

## 2. Experimental

**Figure 1:**
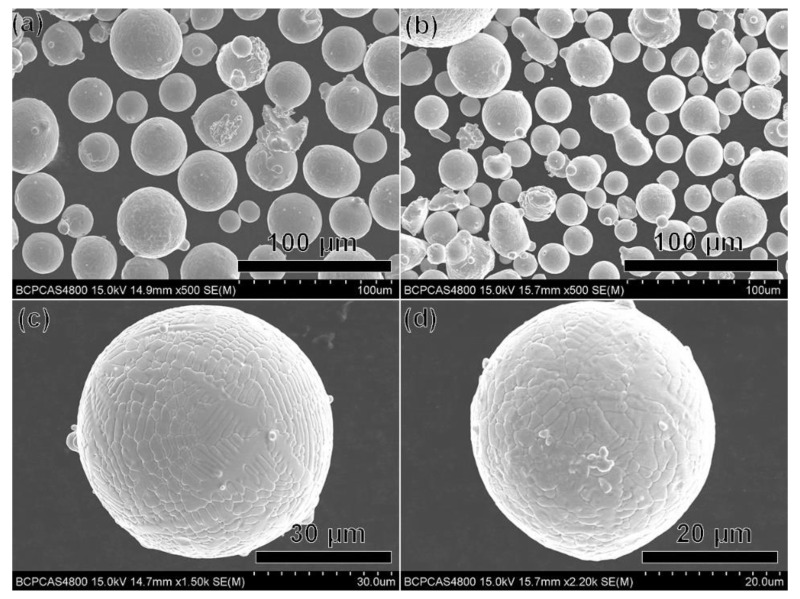
SEM morphologies of the VIGAed powders: (**a**) Haynes 230, (**b**) CRHA, (**c**) Haynes 230, (**d**) CRHA.

The vacuum-induced gas-atomized (VIGAed) Haynes 230 and CRHA powders were supplied by the Shanghai Research Institute of Materials. The nominal chemical compositions of Haynes 230 and the CRHA were listed in Table 1. The powder size was distributed in the range of 15 to 53 μm. SEM morphologies of the Haynes 230 and the CRHA powders are shown in Figure 1. The flow rates of Haynes 230 and the CRHA powders tested through the Hall flow test were 14.65 and 13.37 s/50 g, respectively.

**Figure 2:**
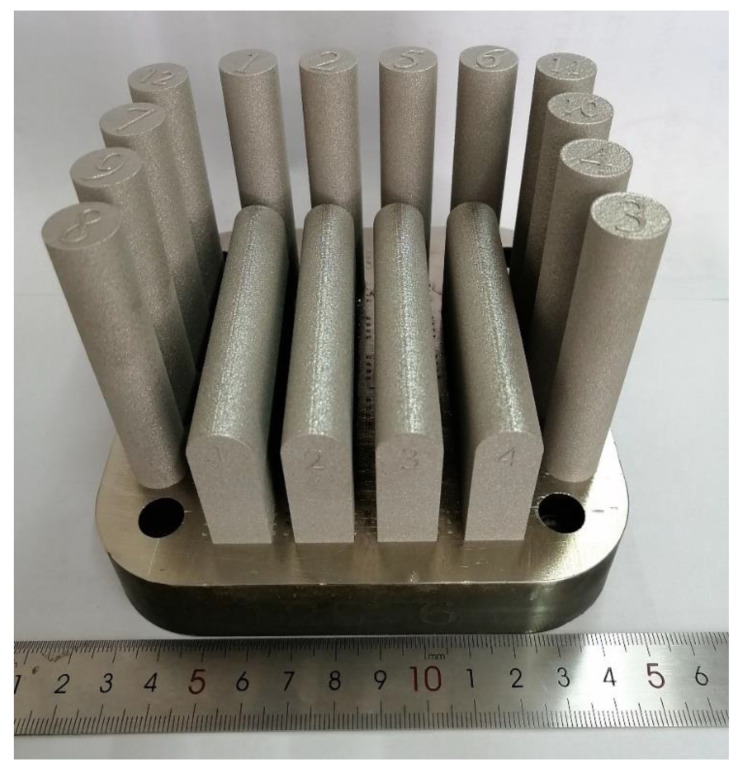
The size and distribution of the SLM processed samples.

The SLM was performed under an Ar atmosphere using an EOS M290 (GmbH Electro Optical System, Munich, Germany) additive manufacturing system. A succession of 30 μm of the GAed powder was applied to build the sample. The scanning space of the laser was 80 μm, the scanning speed was 700 mm/s, and the laser power was 280 W. A meander scan strategy, raster with 67° rotation for each layer, was applied for building all samples. The baseplate was first preheated to 150 °C to avoid stress. The as-built samples of Haynes 230 and the CRHA were distributed as shown in Figure 2. The as-built samples were cut from the baseplate using wire-cut and then submitted to heat treatment. The heat treatment parameters were set as follows: solution, 1180 °C, 8 h, air cooling.

The microstructure of Haynes 230 and the CRHA were characterized using a JEOL 7800 F (JEOL, Ltd., Akishima, Tokyo, Japan) field emission scanning electron microscope (SEM). The SEM was equipped with an Oxford X-MAXN (Oxford Instruments Plc., Abingdon, UK) energy dispersive X-ray spectrometry (EDS, Oxford Instruments Plc., Abingdon, UK) detector and Oxford NordlysMax2 electron backscattered diffraction (EBSD, Oxford NordlysMax2, Oxford, London, UK) detectors. The elements difference of Haynes 230 and the CRHA were investigated using the electron probe microanalysis (EPMA, JEOL JSM-7800F, Tokyo, Japan).

The tensile tests were performed on a Zwick Amsler 100 HFT 5100 tensile testing machine (Zwick Roell Group, Ulm, Germany), with a constant strain of 0.0001 s^−1^ to obtain the ultimate tensile strength (UTS), yield strength (YS) and elongation. Dog bone cylindrical bars with a diameter of 5 mm are used for the tensile test. The distribution and direction of samples for the tensile tests bar are shown in Figure 2. For each type of material, at least 5 specimens were tested to ensure reliability.

## 3. Results

The tensile properties of the CRHA tested at different temperatures were listed in Table 2, while the tensile properties of the Haynes 230 superalloy were also tested for comparison, as displayed in Table 3. The UTS and YS of the vertical direction samples of the CRHA at room temperature were 903 ± 10.5 and 453 ± 6.9 MPa, which is 12 and 33% higher than that of the Haynes 230 superalloy, respectively. The YS and UTS of the vertical direction samples of the CRHA at 1100 °C were 86 ± 3.2 and 116 ± 4.2 MPa, which were approximately 36.5 and 19.5% higher than that of Haynes 230, respectively. In addition, the elongation of the vertical direction samples of the CRHA at 1100 °C was superior to the Haynes 230 superalloy as well, which was increased from 16.9 ± 5.8 to 44.5 ± 9.2. The tensile properties of the vertical direction samples of the CRHA were higher than its counterparts, whether tested at room or high temperature. 

**Figure 3:**
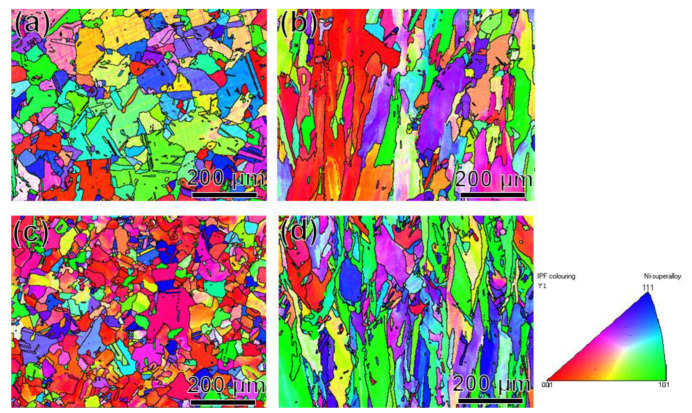
The IPF of the SLM processed Haynes 230 and CRHA. (**a**) was the vertical view of Haynes 230, (**b**) was the horizontal view of Haynes 230, (**c**) was the vertical view of the CRHA, (**d**) was the horizontal view of the CRHA.

To investigate possible reason of the increment in strengths of the CRHA, a rigorous microstructure characterization has been performed. The inverse pole figures (IPF) of Haynes 230 and the CRHA were shown in Figure 3. Figure 3a,c were the vertical view of Haynes 230 and the CRHA, respectively. The legend was given in the lower right corner. Figure 3b,d were the horizontal view of Haynes 230 and the CRHA, respectively. According to Figure 3b,d, the grain morphology of Haynes 230 and the CRHA were columnar grains. The grain morphology of the CRHA was not changed due to the regulation of the elements. However, the orientations of Haynes 230 and the CRHA were different. The orientation of the CRHA was more consistent compared to Haynes 230, as displayed in Figure 3a,c. In addition, the average grain size of Haynes 230 and the CRHA were 36.5 and 28.8 μm, respectively. The grain of the CRHA was a bit smaller than Haynes 230.

**Figure 4:**
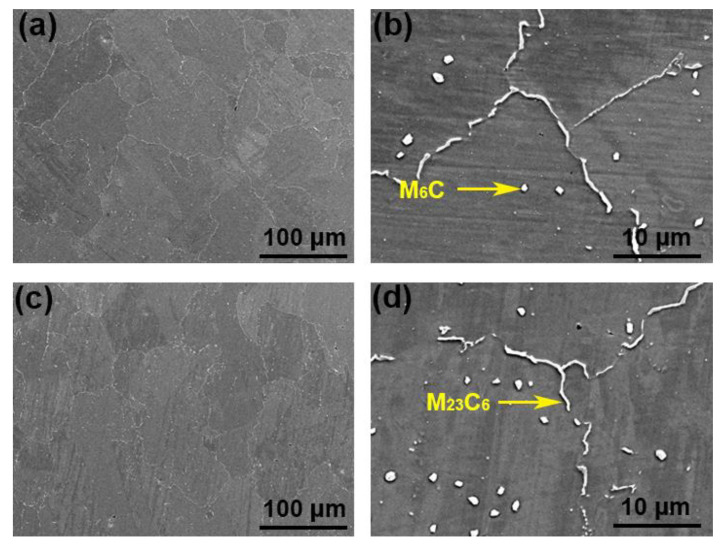
The SEM images of the SLM processed Haynes 230. (**a**,**b**) the vertical view of Haynes 230, (**c**,**d**) the horizontal view of Haynes 230.

**Figure 5:**
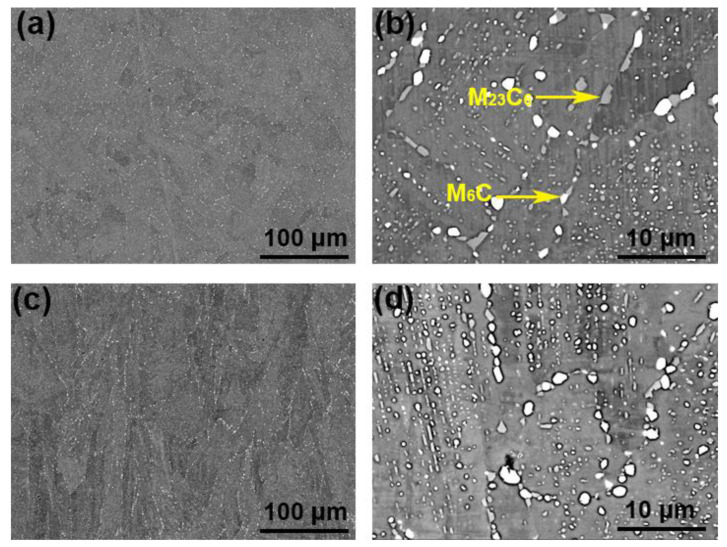
The SEM images of the CRHA. (**a**,**b**) the vertical view of the CRHA, (**c**,**d**) the horizontal view of the CRHA.

**Figure 6:**
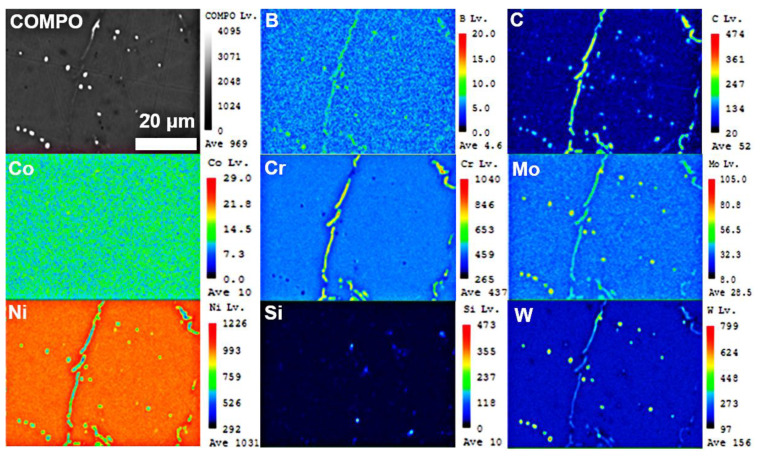
The EPMA results of Haynes 230.

**Figure 7:**
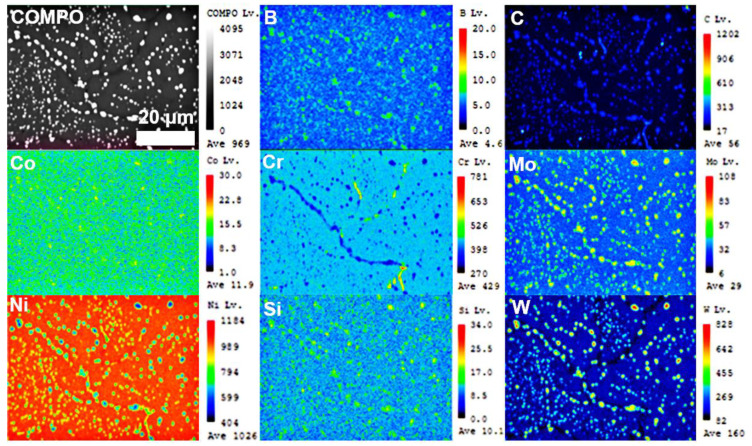
The EPMA results of the CRHA.

Figure 4 and Figure 5 were the microstructure of Haynes 230 and the CRHA captured from different directions. Figure 4 was the microstructure of Haynes 230 after heat treatment. Figure 4a,b show the microstructure captured from the vertical view of the sample and Figure 4c,d were the microstructure captured from the horizontal view of the sample. As can be seen from Figure 4, few strip phases were precipitated along the grain boundaries from the vertical view of Haynes 230. Meanwhile, a few granular particles were also observed inside the grain, which suggests two kinds of phases were precipitated in the Haynes. In the CRHA, the strip phase was hardly observed in both vertical and horizontal directions. There were many particles, bright and gray particles, found in the SEM images, as displayed in Figure 5b,d. The characteristics of the precipitates in Haynes 230 and the CRHA were analyzed using EPMA further. The EPMA results of Haynes 230 and the CRHA were displayed in Figure 6 and Figure 7, respectively. The strip carbides distributed along the grain boundaries were enriched in Cr, Mo and C elements, which was a typical composition of a Cr-rich M_23_C_6_ type carbide in Haynes 230 [23]. Additionally, the W-rich M_6_C type carbide was also observed, as shown in Figure 6. In the CRHA, the strip phases were fewer and enriched in Cr and C elements. Meanwhile, the granular phases were observed as enriched in W, Mo and C elements, as shown in Figure 7. According to the SEM results shown in Figure 4 and Figure 5 and the EPMA results displayed in Figure 6 and Figure 7, it was reasonable to speculate that the carbides were successfully incorporated into Haynes 230.

**Figure 8:**
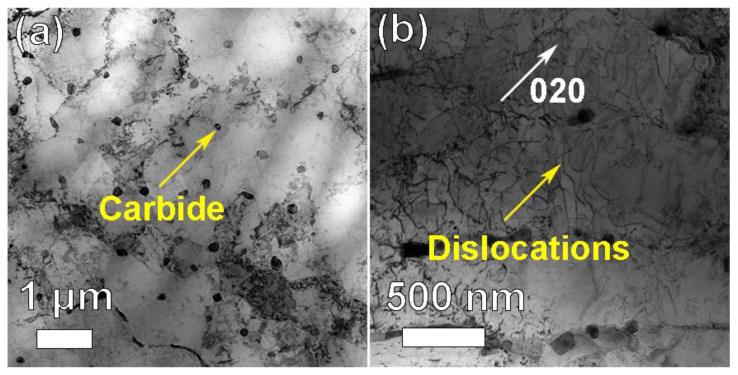
The TEM images of the CRHA. (**a**) bright-field image of the CRHA, (**b**) double weak-beams dark-field image of the CRHA.

**Figure 9:**
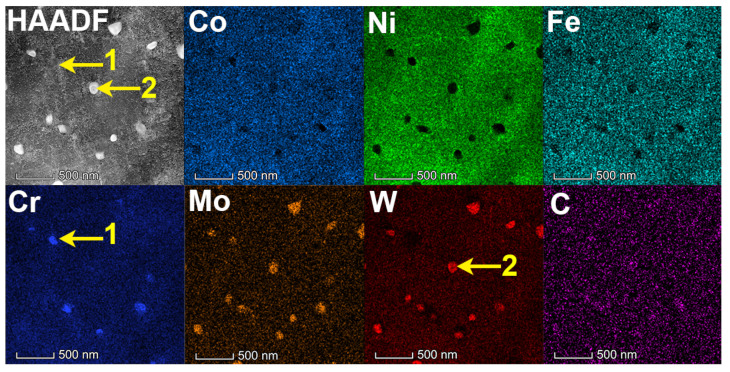
Elements mapping of the CRHA.

**Figure 10:**
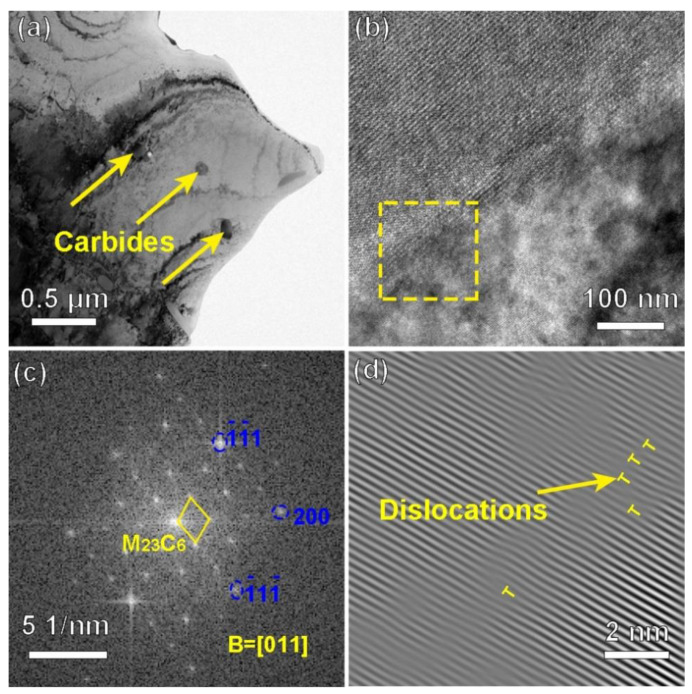
The TEM images showing the carbide in the CRHA. (**a**) the distribution of the carbide, (**b**) high-resolution image of the carbide/γ interface, (**c**) fast Fourier Transform (FFT) of the yellow square area in (**b**,**d**) inverse fast Fourier Transform of (**c**).

To further analyze the microstructure of the CRHA, the TEM observation was performed. Figure 8 was the TEM bright-field (BF) image and weak-beam dark-field (WBDF) image of the CRHA. In Figure 8a, many carbide particles can be founded in the image, as indicated in Figure 8a. As shown in Figure 8a, most of the carbides were granular and distributed uniformly. Meanwhile, some strip carbides were also observed in Figure 8a. In addition, a lot of dislocations were founded in the CRHA, as shown in Figure 8b. Those dislocations may emerge due to the high cooling rate or the mismatch between ceramic particles and metal matrix and result in an increased dislocation density [6]. Figure 9 was the TEM-HAADF image and EDS mapping results of the CRHA. As indicated in Figure 9, two types of carbides were observed in the CRHA, the bright particles rich in W, Mo and C and the gray particles rich in Cr, Mo and C. The chemical compositions of the two type particles were identified using spectral spot scanning and the results are listed in Table 4. According to previous research [24,25,26], those two types of carbides can be identified as M_23_C_6_ (rich in Cr) and M_6_C carbides (rich in W). Figure 10 was the interface between the carbide and the matrix. Figure 10a was the carbides in the CRHA. Figure 10b was the high-resolution TEM image of the interface between the carbide and the matrix. As can be seen from Figure 10b, the interface was clean, no new phase has emerged in the interface. Figure 10c was the Fast Fourier Transform (FFT) image of the yellow square area marked in Figure 10b; Figure 10d was the IFFT image of Figure 10c. In Figure 10c, the diffraction points of the (100) plane of the carbide and diffraction points of (100) plane of the matrix were on a line, which means the (100) plane of the carbide was parallel to the (100) plane of the matrix. Although the (110) plane of the matrix was parallel to the (110) plane of the carbide, some dislocations emerged on the interface between Ni and carbide due to their atomic parameter mismatch, which was consistent with Figure 8b, as shown in Figure 10d.

## 4. Discussion

The strengths of the CRHA were superior to its counterpart Haynes 230. The strengthening factor of the CRHA may affect by factors as follows. The grain size of the CRHA was refined compared to Haynes 230. The average grain size of the CRHA and Haynes were 28.8 and 36.5 μm, respectively. The average grain size of the CRHA was refiner than that of Haynes 230. Hence, the YS of the CRHA will be improved due to the refined grain size and the strengthening value can be estimated using the following Hall–Petch relationship [27]:

(1) $$\sigma_{y}=\sigma_{0}+\frac{k}{\sqrt{d}}\;$$

Here, $\sigma_{y}\;$is the increase in YS due to the refined grain, $\sigma_{0}\;$is the friction stress, *d* is the average grain size and *k* is a constant. According to equation one, the YS of the CRHMC will be increased due to the refined grain size.

For the CRHA, the strengthening of the carbides plays a significant role in the improvement of the YS [28]. The homogeneous dispersed nano-scaled carbides formed in the matrix have a significant effect on the strengths of the CRHA. It is well known that increasing the quantity of the carbides and reducing their average size can strengthen the matrix alloy obviously. The carbides in the CRHA have a high density and their size is very small, which will restrict and impede the moving dislocations more efficiently. The moving dislocations need more energy to circumvent the carbides, resulting in a higher YS of the CRHA. The strengthening effect can be described as follows [29]:

(2) $$\Delta \sigma_{Orowan}=\frac{0.13Gb}{\lambda }ln\left(\frac{d}{2b}\right)$$

Here, the $\Delta \sigma_{Orowan}\;$is the YS increment carbides caused by Orowan strengthening, *G* is the shear modulus, *b* is the Burger vector, *d* is the carbides diameter and λ is particles spacing, as follows:

(3) $$\lambda =d\left[{\left(\frac{1}{2V}\right)}^{1/3}-1\right]$$

Here, *V* is the volume of the carbides. In addition, because of the mismatch between the carbides and the matrix, the thermal stress around the carbides will increase during the cooling process and resulting in plastic deformation in the matrix. As a consequence, some dislocations have emerged in the vicinity of the carbides to the deformation. Hence, a high density of the dislocation in the CRHA is easy to observe around the carbides, as displayed in Figure 8b. The dislocations around the carbides will impede the moving dislocations during the deformation process, resulting in a higher YS of the CRHA. According to the Bailey–Hirsch equation, the increase in the YS can be estimated as follows [27,28]:

(4) $$\Delta \sigma_{d}=M\alpha Gb\rho^{1/2}$$

Here, *M* is the orientation factor, α is a constant, *G* is the shear modulus of the CRHA, *b* is the Burger vector, ρ is the dislocation density. According to equation four, an increased dislocation density will lead to a high YS. In the CRHA, the presence of the carbides not only impedes the moving dislocations but also promotes more dislocations around the carbides to hinder the moving dislocations further. As a result, the strengths of the CRHMC were obviously enhanced compared to the matrix alloy. It is worth pointing out that the strengthening effect of the carbides will be weakened with the increase in the test temperature. At a high temperature, the dislocations that emerged owing to the mismatch between the carbides and the matrix will vanish, resulting in a decreased dislocation density.

## 5. Conclusions

In this study, Haynes 230 and the CRHA were successfully prepared using the SLM process. A detailed characterization of the microstructure of Haynes 230 and the CRHA has been performed following heat treatment. The conclusions were summarized as follows:

1. The SEM and EBSD results reveal that Haynes 230 and the CRHA consisted of elongated grains in the build direction. The average grain size of Haynes 230 and the CRHA were 36.5 and 28.8 μm, respectively.

2. The carbides that emerged in the CRHA were distributed in the matrix uniformly and the carbides were identified as M_23_C_6_ type carbides rich in W, Mo and C and M_6_C type carbides rich in Cr, Mo and C.

3. The increased strengths of the CRHA can be attributed to the synergy strengthening, including refined grain strengthening, Orowan strengthening and dislocation strengthening.

## Figures and Tables

**Table 1:** The normal chemical compositions of the Haynes 230 and the CRHA.

Elements	C	Cr	Co	Al	W	Mo	Ti	Fe	Ni
Content/%	0.08	21.96	0.18	0.36	13.38	2.02	0.014	1.8	Bal.
Content/%	0.15	21.96	0.18	0.36	13.38	2.02	0.014	1.8	Bal.

**Table 2:** The tensile properties of Haynes 230.

Test Temperature	Direction	YS/MPa	UTS/MPa	El/%
RT	Horizontal	336 ± 3.4	769 ± 4.6	37 ± 7.3
	Vertical	340 ± 4.2	800 ± 5.2	58.5 ± 10.5
900	Horizontal	188 ± 3.2	280 ± 4.7	31 ± 8.2
	Vertical	196 ± 4.1	295 ± 3.9	56 ± 9.1
1000	Horizontal	100 ± 5.5	154 ± 4.8	13.5 ± 4.5
	Vertical	99 ± 4.7	152 ± 4.3	17.4 ± 5.3
1100	Horizontal	60 ± 4.1	96 ± 3.3	11.5 ± 3.3
	Vertical	63 ± 4.2	97 ± 2.9	16.9 ± 5.8

**Table 3:** The tensile properties of the CRHA.

Test Temperature	Direction	YS/MPa	UTS/MPa	El/%
RT	Horizontal	453 ± 5.7	733 ± 8.1	9.5 ± 3.1
	Vertical	453 ± 6.9	903 ± 10.5	36 ± 8.6
900	Horizontal	193 ± 5.2	265 ± 6.8	15 ± 4.1
	Vertical	205 ± 4.3	275 ± 7.1	95.5 ± 10.4
1000	Horizontal	128 ± 3.6	175 ± 5.4	6.5 ± 2.2
	Vertical	136 ± 4.1	113 ± 4.9	60.5 ± 9.8
1100	Horizontal	70 ± 2.2	107 ± 4.5	5 ± 2.1
	Vertical	86 ± 3.2	116 ± 4.2	44.5 ± 9.2

**Table 4:** Chemical compositions of carbides (at.%).

Elements	Ni	Co	Cr	Mo	Fe	W	C
Point 1	2.4	3.3	48.5	24.3	4.6	4.1	12.8
Point 2	1.8	2.1	4.5	29.1	2.7	41.6	18.2

## Data Availability

Exclude this statement.
